# Profiling ascidian promoters as the primordial type of vertebrate promoter

**DOI:** 10.1186/1471-2164-12-S3-S7

**Published:** 2011-11-30

**Authors:** Kohji Okamura, Riu Yamashita, Noriko Takimoto, Koki Nishitsuji, Yutaka Suzuki, Takehiro G Kusakabe, Kenta Nakai

**Affiliations:** 1 Human Genome Centre, Institute of Medical Science, University of Tokyo, Tokyo, Japan; 2 Centre for Informational Biology, Ochanomizu University, Tokyo, Japan; 3 Department of Life Science, Graduate School of Life Science, University of Hyogo, Hyogo, Japan; 4 Department of Biology, Faculty of Science and Engineering, Konan University, Kobe, Japan; 5 Department of Medical Genome Sciences, Graduate School of Frontier Sciences, University of Tokyo, Kashiwa, Japan

## Abstract

**Background:**

CpG islands are observed in mammals and other vertebrates, generally escape DNA methylation, and tend to occur in the promoters of widely expressed genes. Another class of promoter has lower G+C and CpG contents, and is thought to be involved in the spatiotemporal regulation of gene expression. Non-vertebrate deuterostomes are reported to have a single class of promoter with high-frequency CpG dinucleotides, suggesting that this is the original type of promoter. However, the limited annotation of these genes has impeded the large-scale analysis of their promoters.

**Results:**

To determine the origins of the two classes of vertebrate promoters, we chose *Ciona intestinalis*, an invertebrate that is evolutionarily close to the vertebrates, and identified its transcription start sites genome-wide using a next-generation sequencer. We indeed observed a high CpG content around the transcription start sites, but their levels in the promoters and background sequences differed much less than in mammals. The CpG-rich stretches were also fairly restricted, so they appeared more similar to mammalian CpG-poor promoters.

**Conclusions:**

From these data, we infer that CpG islands are not sufficiently ancient to be found in invertebrates. They probably appeared early in vertebrate evolution via some active mechanism and have since been maintained as part of vertebrate promoters.

## Background

Among the 16 DNA dinucleotides, the CpG dinucleotide is unique in terms of its frequency in genomic sequences. This most probably results from the DNA methylation system because the DNMT1 and DNMT3 families of the deuterostomes, such as echinoderms and chordates, predominantly target the 5 position of cytosine residues only in the CpG dinucleotide [1]. Because the deamination of 5-methylcytosine is not recognized by the DNA repair mechanisms, CpG is rapidly mutated to TpG or to its complementary dinucleotide CpA [2]. Therefore, deuterostome organisms, except for *Oikopleura dioica *[3], display a globally reduced frequency of the CpG dinucleotide compared with its expected frequency calculated from actual numbers of guanine and cytosine residues [4,5]. Interestingly, they also display skewed distributions of the CpG dinucleotide across their genomes, so that their genomes contain CpG-poor and CpG-rich domains [6,7]. In amphibians, avians, and mammals, the CpG-rich domains are much shorter than the CpG-poor domains and are generally known as CpG islands [8].

CpG islands are good markers of some classes of genes because they are often linked to the promoters of those genes [9]. In most cases, CpG islands escape DNA methylation, which suppresses gene expression in general, in almost every tissue [10] and function as part of the gene promoter [11]. Hence, CpG islands tend to be related to ubiquitously or broadly expressed genes, whereas promoters that lack a CpG island are involved in the spatiotemporal regulation of the genes [12]. It is important to note that mammalian promoters can be thus divided into the two distinct classes, not only structurally but also functionally. In the human genome, CpG-rich promoters or CpG island promoters are dominant, occurring more than twice as often as CpG-poor promoters [13,14].

As anticipated for a vertebrate taxon, CpG island promoters were indeed experimentally identified in fish by an analysis of transcription start sites (TSSs) [15]. The presence of two classes of promoters in fish, amphibians, reptiles, avians, and mammals has since been confirmed *in silico *[16]. In that study, the authors analysed the distributions of the normalized CpG contents (the ratio of the observed CpG number to the expected CpG number, called the "CpG score" hereunder) of the promoter sequences in six vertebrate genomes and showed bimodal distributions for all of them. Furthermore, the structural bimodality was shown to correspond to functionally distinct classes of genes. The authors also analysed three invertebrate promoters, of one sea urchin and two ascidian (sea squirt) species, and found unimodal distributions of high CpG scores, unlike the distributions observed in the vertebrate promoters. This led them to propose that the vertebrate promoter classes differentiated at an early stage of vertebrate evolution, with global DNA methylation and subsequent deamination. This is basically consistent with the formerly accepted evolutionary hypothesis of CpG islands [17,18].

If this hypothesis is true, do the non-vertebrate deuterostomes (*e.g.* echinoderms, lancelets, and ascidians) have CpG islands in their genomes? Currently, the presence of CpG islands in invertebrate animals is unclear. It is possible to apply any criteria that define a CpG island to their genomic sequences and identify some islands. Nevertheless, we were interested in determining whether there are CpG island-like sequences in invertebrate genomes that are associated with transcription initiation, and how and when these sequences appeared during evolution.

To address this issue, we identified the TSSs of *Ciona intestinalis* by a combination of the oligo-capping method [19] and massive-scale cDNA sequencing (RNA-seq, specifically TSS-seq) [20]. The widely used model organism *C. intestinalis* is an ascidian tunicate, which although an invertebrate, is most closely related to the vertebrates [21]. Although the ascidian evolved from the last common ancestor of the ascidians and vertebrates, it can be presumed to retain many more features of the ancestral organism than do extant vertebrates. It is well known that the enrichment of the CpG dinucleotides in CpG island promoters is maximum in TSSs [12,13], so TSSs constitute candidate regions in which CpG island promoters or CpG island-like sequences might occur in the invertebrate genome. Incidentally, this approach that targets TSSs also circumvents the confusion arising from CpG-rich sequences that are indifferent to transcription initiation. In the computational study mentioned above, promoter regions were defined using the RefSeq database, which is a curated collection of publicly available nucleotide sequences [16]. It is likely that many of the cDNA entries are truncated or incomplete at the 5’ end which makes the definition of their promoter regions unreliable. More importantly, the TSSs of approximately half of all ascidian genes can hardly be determined because of mRNA 5’-leader *trans*-splicing [22-24]. The 5’ ends of those primary transcripts, termed the outron, are discarded via the *trans*-splicing reaction. This fact is easily exemplified by downstream operonic genes, which are resolved from their primary transcripts by *trans*-splicing [25]. Although it is almost impossible to know TSSs of them, it is essential to be distinguished from non-*trans*-spliced genes and to know the most 5' end position of the processed transcripts. Analyzing these data, we determined the structural features of the ascidian promoters and compared them with human promoters to identify and characterize their similarities and differences. To extend our understanding of gene regulation in higher eukaryotes, we undertook to clarify the origin of CpG islands and the two classes of vertebrate promoters.

## Results

In this study, we chose *C. intestinalis* embryos at the mid-tailbud stage (Additional file 1: Figure S1) for the genome-wide identification of TSSs. Since whole embryos still retaining the notochord contain a wide range of cell types, we may cover a large part of ascidian promoters. Total RNA was extracted from embryos and was subjected to oligo capping in which the 5’ cap of the mRNA was replaced with a synthetic RNA oligonucleotide (see Methods). After cDNA synthesis and subsequent PCR, we undertook massively parallel sequencing using the Illumina Genome Analyzer. We obtained two data sets containing fragments of different lengths 36 nt or 48 nt. Because we read the sequences from the 3’ end of the RNA oligonucleotide, all the sequences obtained should start with GG at their 5’ ends (see Methods). We recovered only the reads that started with GG, but then trimmed the GG from those. Although the genic sequences were trimmed by two nucleotides, this protocol eliminated dubious sequences that do not start with the dinucleotide. We also eliminated sequences containing undetermined nucleotides other than T, C, A, and G, yielding 4,247,902 reads of 34 nt and 4,770,608 reads of 46 nt. To detect the spliced leader (SL) of *C. intestinalis*, we considered, in addition to the canonical 16-nt sequence, all similar sequences, allowing a 1-nt mismatch or indel and some previously reported variants [24]. The 34-nt data set consisted of 1,849,849 non-*trans*-spliced and 2,398,053 *trans*-spliced reads and the 46-nt data set consisted of 2,052,230 non-*trans*-spliced and 2,718,378 *trans*-spliced reads. Even if some SL-related 5’ mRNA sequences escaped from being detected by this process, it is unlikely such reads would map to the genome in the following step. Mapping or alignment to the KH assembly [25] was performed as described in the Methods. Sequences that mapped to more than one locus (multiple hits) were not considered further. The numbers of mapped 34-nt and 46-nt reads were 1,017,283 (non-*trans*-spliced), 1,932,570 (*trans*-spliced), 939,092 (non-*trans*-spliced), and 1,237,720 (*trans*-spliced), respectively. Because the original 5’-segment of a pre-mRNA is discarded during the *trans*-splicing reaction, mature *trans*-spliced mRNAs do not contain the initial segment of the primary transcript and therefore lack the information required to precisely identify TSS [22]. Therefore, we decided to mainly examine non-*trans*-spliced reads to provide valid data for the promoter analyses presented here. The genomic positions to which the 5’ ends of the reads were aligned were defined as TSSs. The read counts were converted to values in parts per million (ppm) for transcript abundance estimation and normalization, and both of the short and long data sets were merged. The TSSs, which are generally scattered around a promoter region [26], were then clustered into 100-bp bins to define each promoter. In other words, two reads located more than 100 bp apart without any other reads between them were considered to be regulated by two separate promoters [26]. In this clustering process, TSSs represented by reads occurring at less than 0.5 ppm were not considered. However, once promoters were defined, all the TSSs in the bins were counted to estimate the abundance of transcripts from each cluster. Because we can assume that every cell contains approximately one million mRNA molecules, we can consider the values in ppm as copy numbers of the transcripts in a cell [27]. We set a threshold of 1.0 ppm to exclude transcriptional noise. As a result, we obtained 6312 and 8753 promoters for non-*trans*-spliced and *trans*-spliced genes, respectively, that could be considered active in the tailbud embryos. The most frequent TSS in each promoter (and if there were several, the most upstream one) was selected as its representative TSS. If the corresponding genes were found in the KH gene model [25], the gene names were also tabulated (Additional file 2: Tables S1 and S2). Note that one gene can have several alternative promoters.

**Figure 1:**
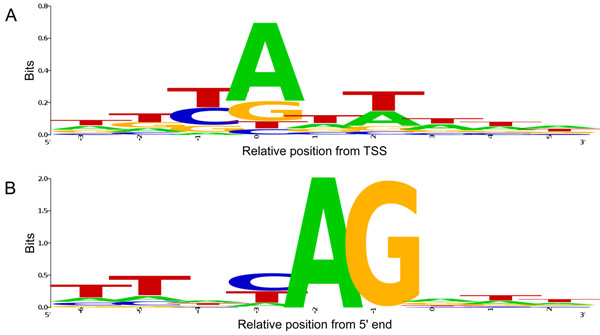
**Sequence logo around the ascidian transcription start sites.** (A) All the 6312 promoter sequences were aligned around the representative TSSs with consideration of their transcriptional orientation. The pyrimidine-purine (YR) consensus was also observed in the ascidian genome. The TSS is located at position 0. (B) A similar alignment of the 5' ends of the first exons of all the 8753 *trans*-spliced transcripts is also shown. In this case, the position 0 means the most 5' end of the exons. Splice acceptor sequences, which are replaced with SLs in the *trans*-splicing reaction, can be observed. The whole replaced sequences are also known as outrons.

The initiator (Inr) motif, which spans the TSS, is the most commonly occurring sequence motif observed in metazoans [28]. Its consensus sequence between mammals and fruit fly is pyrimidine-purine (YR), where R corresponds to the exact TSS [29]. By aligning core promoter sequences of all the 6312 non-*trans*-spliced transcripts with consideration of their orientation, we confirmed that the ascidian promoters also follow the YR consensus, suggesting that the sequence processes described above are plausible (Figure 1). In this figure, all the representative TSSs are aligned at position 0. The next positions upstream and downstream are designated -1 and +1, respectively. This notation is used in the rest of the present paper. Another alignment of all the 8753 *trans*-spliced transcripts is also shown. In this case, however, the position 0 means the most 5' end of the transcripts after removing SLs.

**Figure 2:**
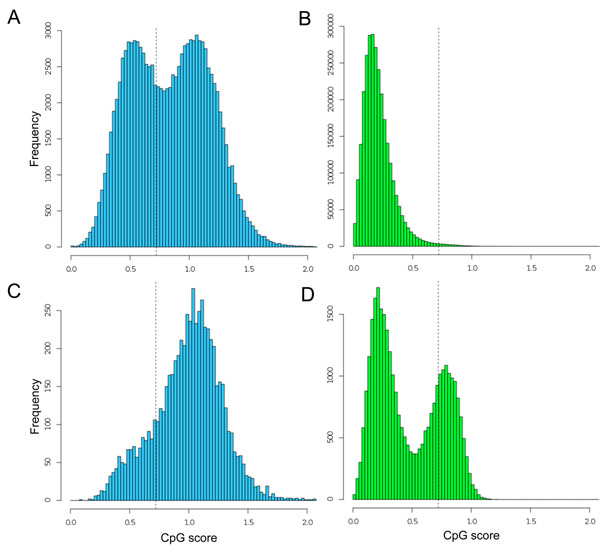
**Distributions of the CpG score frequencies in 1-kb genomic fragments and promoters.** The histograms show the distributions in the ascidian genome (A), the human genome (B), ascidian promoters (C), and the promoters of human protein-coding genes (D). Vertical dotted lines indicate a CpG score of 0.721, around which human CpG island promoters are dispersed [5]. The distributions of the overall genome scores differ between human and ascidian (unimodal vs. bimodal, respectively), but the distributions of the promoter scores of the human and ascidian sequences differ obversely (bimodal vs. unimodal, respectively).

We then examined the genome-wide distributions of the CpG scores in both the whole genome and the promoters of non-*trans*-spliced transcripts, using a sliding window of 1 kb. To compare them with the corresponding vertebrate distributions, we performed the same analysis using the human genome (Figure 2). We defined a sequence fragment from -499 to +500 as a promoter. A similar analysis of the CpG-score distributions has already been reported [16]. Although the definitions of the promoter sequences differ in these studies, we obtained fundamentally identical results. The human genome is globally methylated and CpG dinucleotides occur in bulk at only one-fifth of the expected frequency [17]. In contrast, the ascidian genome contains approximately equal amounts of methylated and unmethylated regions, which may have resulted in CpG-poor and CpG-rich sequences, respectively [7,14]. Intriguingly, the ascidian and human promoters show unimodal and bimodal distributions, respectively. The latter distribution indicates that the human has two classes of promoters, CpG-poor and CpG-rich. The CpG-rich promoters can be considered to contain a CpG island. In contrast, the ascidian promoters generally tend to have high CpG scores and exhibit a unimodal distribution. This observation led to the hypothesis that human CpG-poor promoters emerged with the deamination of methylated CpG dinucleotides in CpG island promoters [16]. Using our experimental data, we intended to substantiate this idea and define the CpG islands in the invertebrate genome.

**Figure 3:**
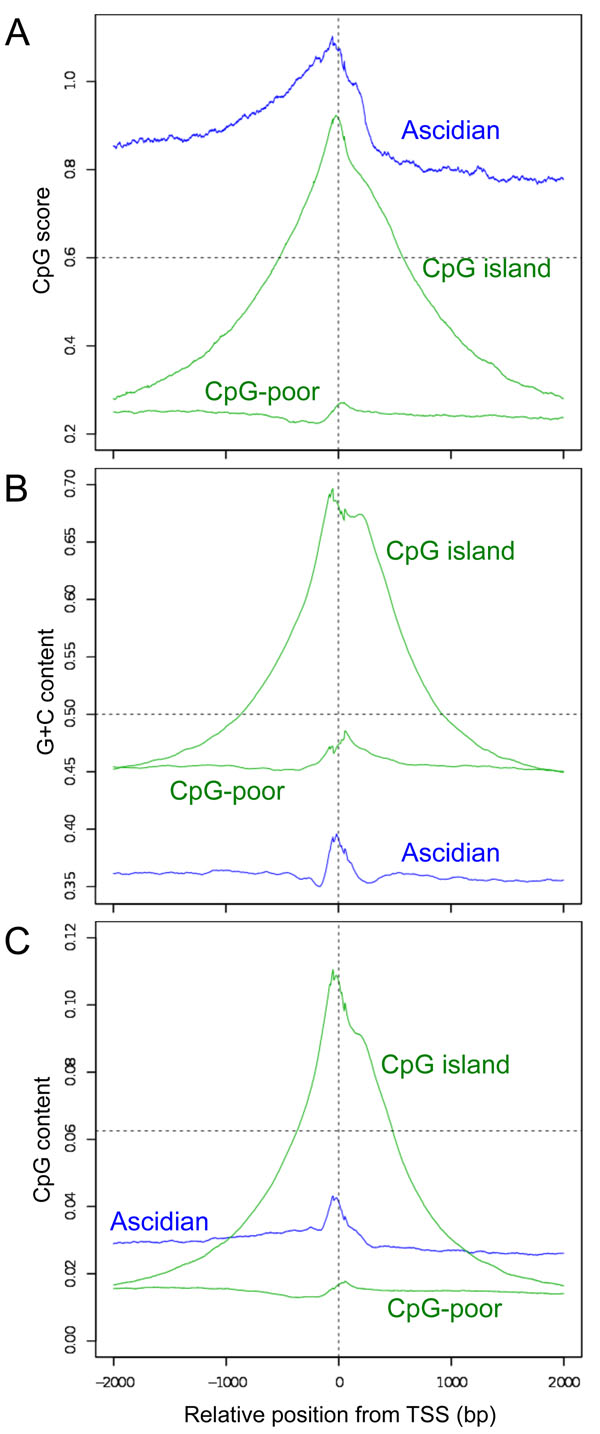
**Changes in the CpG scores (A), G+C contents (B), and CpG contents around the TSS.** The local CpG score, G+C content, and CpG content at each position within a 4-kb region, with a moving window size of 100 bp, were averaged for the ascidian, human CpG-poor, and CpG island promoters. The horizontal axes indicate the position relative to the representative TSS. Horizontal dotted lines indicate the conventional criteria for vertebrate CpG islands (A, B) and the expected content of each dinucleotide, 1/16 (C).

We excised 4-kb promoter sequences (2 kb upstream and 2 kb downstream from each representative TSS) of the ascidian non-*trans*-spliced, and human CpG-poor, and CpG-rich promoters, and aligned them with consideration of the transcriptional orientation to determine the overall changes in the CpG scores and G+C contents in the vicinity of the TSSs (Figure 3). We used Database of Transcription Start Sites (DBTSS) to select the human promoter sequences [27]. The methodological details such as grouping human CpG-poor and CpG-rich promoters are described in the Methods. Our results confirmed that the ascidian promoters tended to have high CpG score and G+C contents around TSS, as was observed in the human promoters. However, judging from the heights and extents (widths) of the peaks around the TSSs, the ascidian promoters seem more similar to the human CpG-poor promoters than to the human CpG island promoters (Figure 3B). Although the ascidian TSSs exhibited quite high CpG score, this fact does not necessarily mean that they have high frequency of the CpG dinucleotide (Figure 3A). The low content of G+C underestimated the expected number of CpG, which in result increased the ratio of the observed over expected numbers of the dinucleotide, *i.e.* CpG score. Hence, we defined "CpG content" to show its plain density (see Methods) and drew the changes (Figure 3C). The heights and extents were comparable between the ascidian and CpG-poor promoters and their contents were regularly lower than the expected content for any dinucleotide, 0.0625 or 1/16. In addition to CpG, we also analysed the changes in all the other dinucleotide scores in the vicinity of the TSSs (Additional file 3: Figure S2). Distinct features were also observed at the TSSs for all these dinucleotide scores. This information may possibly be used to predict the locations of promoters and their corresponding genes.

**Figure 4:**
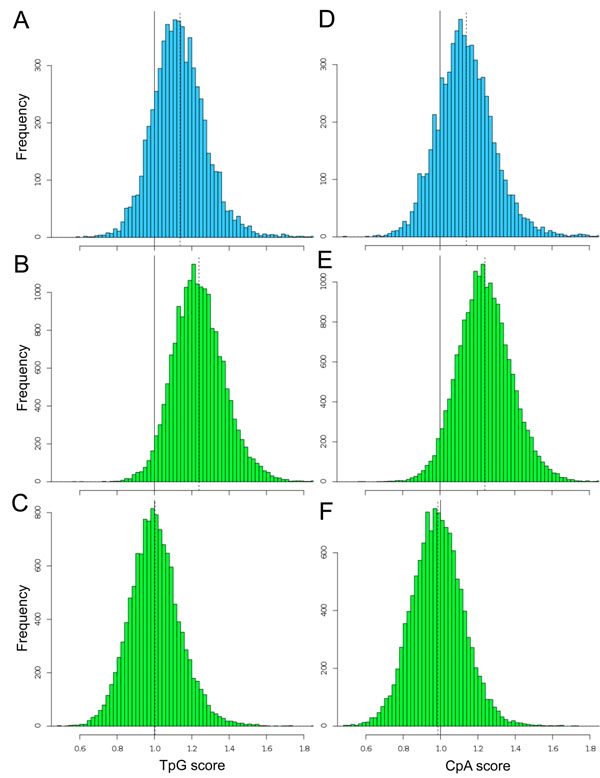
**Distributions of TpG and CpA scores in 1-kb promoters.** The left histograms show the distributions of TpG scores in the ascidian (A), human CpG-poor (B), and CpG island promoters (C). The right histograms show the distributions of CpA scores in the ascidian (D), human CpG-poor (E), and CpG island promoters (F). Vertical dotted lines indicate the positions of mean, *i.e.* 1.14, 1.24, 1.00, 1.14, 1.24, and 0.99 for (A)-(F), respectively The score 1.0 means that the observed and expected numbers of the dinucleotide are equal, suggesting no methylation effect in this case.

Among the dinucleotides, the local frequencies of TpG and CpA can be used as indicators of DNA methylation levels [4]. We calculated the TpG and CpA scores for 1-kb promoter sequences and charted their distributions for the three classes of promoters (Figure 4). All the six histograms showed a unimodal bell-shaped distribution, *e.g. p* < 10^-15^ by Kolmogorov-Smirnov test for Figure 4A, indicating that they were formed by promoters having homogeneous characters in terms of the dinucleotide scores. Whereas the distributions of the human CpG island promoters are centered at the value of 1.0, the distributions of the ascidian and CpG-poor promoters are shifted to higher-score regions, where observed numbers of the deaminated dinucleotides are larger than their expected numbers. It is more likely that deamination of CpG sites are common. The high frequency of deamination in the ascidian and CpG-poor promoters suggests that these regions are relatively methylated unlike CpG islands. Because mutations in somatic cells have not been transmitted evolutionarily, what we observed here is the result occurred in germ line. The DNA methylation could be tissue-, stage-, or cell-type-specific and play a role in spatiotemporal gene regulation.

**Figure 5:**
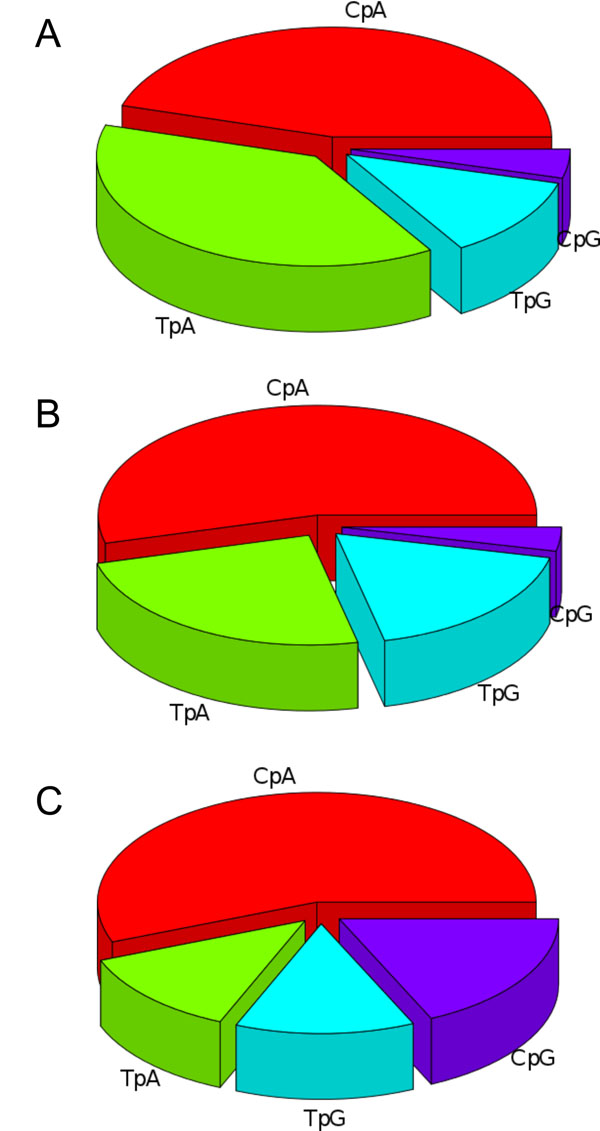
**Usage of the four YR dinucleotides at the TSS.** The proportional use of each YR dinucleotide at the representative TSSs was calculated for the three promoter groups: ascidian (A), human CpG-poor (B), and human CpG island promoters (C). Dinucleotides other than CpA, CpG, TpA, and TpG were ignored in this analysis. Whereas CpG is the second most often used dinucleotide in human CpG-rich promoters, the dinucleotide is used least in ascidian and human CpG-poor promoters.

Lastly, we examined the usage of the four YR dinucleotides (CpA, CpG, TpA, and TpG) at the YR-consensus sites (positions -1 and 0). This analysis was performed using representative TSSs, which have a one-to-one correspondence with promoters. As noted above, these dinucleotides are preferentially used as TSSs in a wide range of animals [29]. However, the frequencies of the dinucleotides are not equivalent (Figure 5). CpA is the most commonly observed as the representative TSS in both ascidian and human genomes. The second preference is for CpG in human CpG island promoters. The usages of CpG are 4.2%, 3.5%, and 18.1% in the ascidian, human CpG-poor, and CpG island promoters, respectively. Although the ascidian promoters tended to exhibit high CpG scores (Figure 2), CpG seems to be used rarely as the transcription initiation point.

## Discussion

The CpG island promoters seen in vertebrates are believed to have emerged from the deamination of other regions [17]. Therefore, it is plausible that the appearance of the two classes of vertebrate promoters is also a consequence of deamination, following the global DNA methylation that occurred early in vertebrate evolution [16,30]. Specific sequence motifs that function as transcription factor binding sites might have retained some CpG-rich sequences from the methylation and mutation to form CpG island promoters [31-33]. To confirm this hypothesis, we used a large-scale experimental approach to identify the TSSs of *C. intestinalis*. On the basis of our TSS information, we then examined the ascidian promoter sequences. The fact that the CpG scores, *i.e.* the ratios of the observed CpG number to the expected CpG number, tended to be quite high in the vicinity of the ascidian TSSs led us speculate CpG island promoters [16]. However, it had to be noted that the G+C and CpG contents are low. When we applied the most conventional and conservative CpG island definition [8] to the promoters, only 3.5% (223 out of the 6313 promoters) meet the criteria. This is attributable to the fact that the ascidian G+C content, approximately 0.36, is much lower than the G+C criterion of 0.5 (Figure 3B). Even at TSSs, the average ascidian G+C content is approximately 0.4 at the most. Besides, the ascidian CpG score is much higher than the criterion of 0.6 (Figure 3A). If we try to define new criteria for the ascidian genome, the difference in the values for the TSSs and background sequences is much smaller than that observed for the human genome. The unique feature of the non-vertebrate deuterostome genomes, *i.e.* the presence of comparable amounts of CpG-poor and CpG-rich domains [7], also hinders us in defining CpG islands in these animals.

Contrary to our initial expectation, we failed to identify CpG island-like promoters in the invertebrate genome. Instead, we found that the general features of ascidian promoters are similar to those of CpG-poor vertebrate promoters rather than to CpG island promoters. It is reasonable to consider CpG-poor promoters more ancient because they are found in a wide variety of eukaryotes [29]. Conversely, CpG island promoters must have appeared in an early stage of vertebrate evolution, derived by some mechanism, and have been adopted as important *cis* regulatory elements in descendant species. Because the CpG score is just the ratio of the observed to the expected numbers of dinucleotides, a high score does not necessarily mean a high frequency. We defined and used "CpG content", which showed a substantially different feature from CpG score in the ascidian genome (Figure 3C). Note that the CpG score and CpG content profiles are dissimilar and similar in the ascidian and human genomes, respectively. The CpG content will also be important to scrutinize genomes especially of various animals other than mammals. It is unlikely that the conventional CpG island definitions using only CpG score, G+C content, and length function in invertebrate genomes. Because the deamination of methylated CpG sites cannot explain the substantial increase in the CpG and G+C contents in the vicinity of vertebrate TSSs, we must search for and examine active mechanisms that may have given rise to CpG islands. The biased gene conversion [18,34], the condensation of CpG-rich protein-coding sequences by retrotransposition [35], and the expansion of elements containing the CpG dinucleotide [36] are potential molecular mechanisms. The fact that CpG islands are not conserved satisfactory among species [8] may indicate that CpG island loss and gain are active phenomena, occurring up to the present time, even in extant vertebrates.

The number of *C. intestinalis* genes is reported to be 15,254 in the KH gene model [25]. Whereas series of operonic genes have single promoters, alternative promoters have been reported for a large number of genes. The number of all RNA polymerase II promoters, including those of non-coding transcripts, may exceed 20,000. This study targeted the promoters that are active in the embryos. Although we believe that the 6312 promoters analysed here may well represent most of them, we eagerly await techniques with which to identify the TSSs of *trans*-spliced genes. Utilizing our data, the TSS of the *TnI* gene was recently identified as the first case for *Ciona trans*-spliced genes [36]. CpG island promoters cannot be seen at least for this gene.

## Conclusions

We have experimentally identified and characterized ascidian promoter sequences as the primordial type of vertebrate promoter. As far as we know, this is the first case for non-vertebrate deuterostomes. The sequences near TSSs tend to exhibit high CpG score and high G+C content, but their level and extent are actually restricted. Furthermore, the promoter sequences seem to be at least partially methylated. It is unlikely that they were the original type of vertebrate CpG island promoters. Rather than global methylation and subsequent deamination, some active mechanisms and maintaining mechanisms have presumably been required to form such a long and CpG-condensed region in vertebrate animals.

The genomes of more than 50 vertebrate species have been sequenced and even more genomes will be sequenced in the future [38]. Now that an ascidian genome has been shown to lack CpG islands that function in promoter sequences, our curiosity is directed to primitive vertebrates, such as agnathans. It could be superficial to make a strong conclusion at this point. The searching for primitive organisms with CpG island promoters in order to determine the origin of CpG islands will certainly extend our understanding of the sophisticated roles of DNA methylation in higher eukaryotes [39-41].

## Methods

### RNA extraction, oligo capping, and RNA-seq with the Illumina Genome Analyzer

More than 200 μg of total RNA was isolated from whole mid-tailbud-stage ascidian embryos (12-hour-old embryos), using ISOGEN (Nippon Gene) according to the manufacturer’s protocol. The RNA was subjected to oligo-capping method [19]. In short, after successive treatments with bacterial alkaline phosphatase (TaKaRa) and tobacco acid pyrophosphatase (Ambion), the treated RNA was ligated to an RNA oligonucleotide with the sequence 5’- AAU GAU ACG GCG ACC ACC GAG AUC UAC ACU CUU UCC CUA CAC GAC GCU CUU CCG AUC UGG -3’ using T4 RNA ligase (TaKaRa). After treatment with DNase I, the poly(A)^+^ RNA was selected and used as the template for the first-strand cDNA synthesis with the primer 5’- CAA GCA GAA GAC GGC ATA CGA NNN NNN C -3’. The cDNA was then used as the template for PCR with the primers 5’- AAT GAT ACG GCG ACC ACC GAG -3’ and 5’- CAA GCA GAA GAC GGC ATA CGA -3’. The products were size fractionated by polyacrylamide gel electrophoresis. Approximately 1 ng of the 150-200-bp fraction was used for the sequencing reactions on the Illumina Genome Analyzer (Solexa). Both 36-cycle and 48-cycle sequencing reactions were performed on the same samples. The DNA sequences have been deposited in [DDBJ Sequence Read Archive: DRA000156].

### Sequence data analysis

Illumina Pipeline (GAPipeline 1.0) was used to extract the sequenced reads from the image data. The spliced leaders (SLs) in the *trans*-spliced sequences were replaced with splice acceptor sequence “ag” for the subsequent mapping. The sequences were aligned to the KH assembly [25] using SeqMap [42] for the 36-cycle reads, or to BLAT [43] for the 48-cycle reads, because of the high rate of *cis*-splicing. Because of the highly polymorphic genic features of this organism [44], we used a 90% match criterion, including insertions and deletions. If the 5’ end of a read was not aligned to the genome, the read was eliminated from the analysis. Multiple hits were removed, and only single best hits were considered for the subsequent analyses. Sequence logos were drawn with WebLogo 2.8.2 (http://weblogo.berkeley.edu/). The CpG score was defined as *CpG* * *N* / *C* / *G* with *C*, *G*, *CpG*, and *N* observed numbers of C, G, and CpG and the fixed window size, respectively. The CpG content defined in the present study was *CpG* / (*N* - 1). The assembly used for the human genome was UCSC hg18. To select human CpG-poor (CpG score < 0.5) and CpG-rich promoters (CpG score > 0.6), we used DBTSS 6.0 (http://dbtss.hgc.jp/) and calculated CpG scores in 200-bp regions around representative TSSs [45]. The analysis was limited to protein-coding genes, but all the alternative promoters deposited in the database were included (out of all 101,436 promoters, 32,122 were for protein-coding genes). The numbers of CpG-poor and CpG-rich promoters were 18,034 and 12,493, respectively. Dinucleotides other than pyrimidine-purine (YR) were not considered in the analysis of the usage of the YR motif. The total numbers of YR motifs at TSSs were 3,610, 8,162, and 8,610 for ascidian, human CpG-poor, and human CpG island promoters, respectively. All the sequence analyses were performed with Perl scripts, which are available upon request.

## List of abbreviations used

TSS: transcription start site; SL: spliced leader

## Competing interests

The authors declare that they have no competing interests.

## Authors' contributions

K.O. conceived of the study, designed the study, and drafted the manuscript; R.Y. helped to analyze data; N.T, K.N., and T.G.K. prepared *Ciona *embryos and extracted RNA; Y.S. performed sequencing; and K.N. participated in the coordination of the study.

## Supplementary Material

Additional file 1 ***Ciona intestinalis* embryos at mid-tailbud stage (Figure S1)** A photo of two *C. intestinalis* embryos at mid-tailbud stage, taken 12 hours after fertilization. Click here for file

Additional file 2 **Summary of the TSS-seq experiments (Tables S1 and S2)** Quantitative data for the 6312 non-*trans*-spliced and 8753 *trans*-spliced transcripts which were annotated by the nearest genes. Click here for file

Additional file 3 **Changes in dinucleotide scores in the vicinity of TSSs (Figure S2)** The score changes are shown for ascidian promoters (A), human CpG-poor promoters (B), and human CpG-rich promoters (C). Click here for file
