# Supplementary material for: Profiling ascidian promoters as the primordial type of vertebrate promoter
Source: BMC Genomics. 2011 Nov 30;12(Suppl 3):S7. doi: 10.1186/1471-2164-12-S3-S7 (PMC3333190; doi:10.1186/1471-2164-12-S3-S7)
Supplement: Additional file 1 — Ciona intestinalis embryos at mid-tailbud stage (Figure S1) A photo of two C. intestinalis embryos at mid-tailbud stage, taken 12 hours after fertilization. [file 1471-2164-12-S3-S7-S1.pdf]

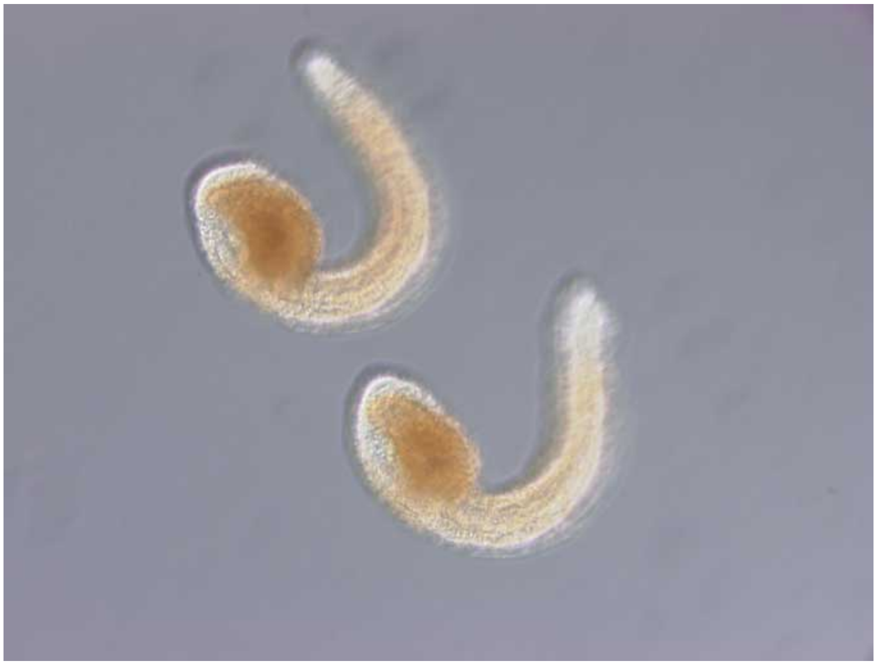

Fig. S1. *Ciona intestinalis* embryos at mid-tailbud stage.
This picture was taken 12 hours after fertilization.
