# Supplementary material for: Profiling ascidian promoters as the primordial type of vertebrate promoter
Source: BMC Genomics. 2011 Nov 30;12(Suppl 3):S7. doi: 10.1186/1471-2164-12-S3-S7 (PMC3333190; doi:10.1186/1471-2164-12-S3-S7)
Supplement: Additional file 3 — Changes in dinucleotide scores in the vicinity of TSSs (Figure S2) The score changes are shown for ascidian promoters (A), human CpG-poor promoters (B), and human CpG-rich promoters (C). [file 1471-2164-12-S3-S7-S3.pdf]

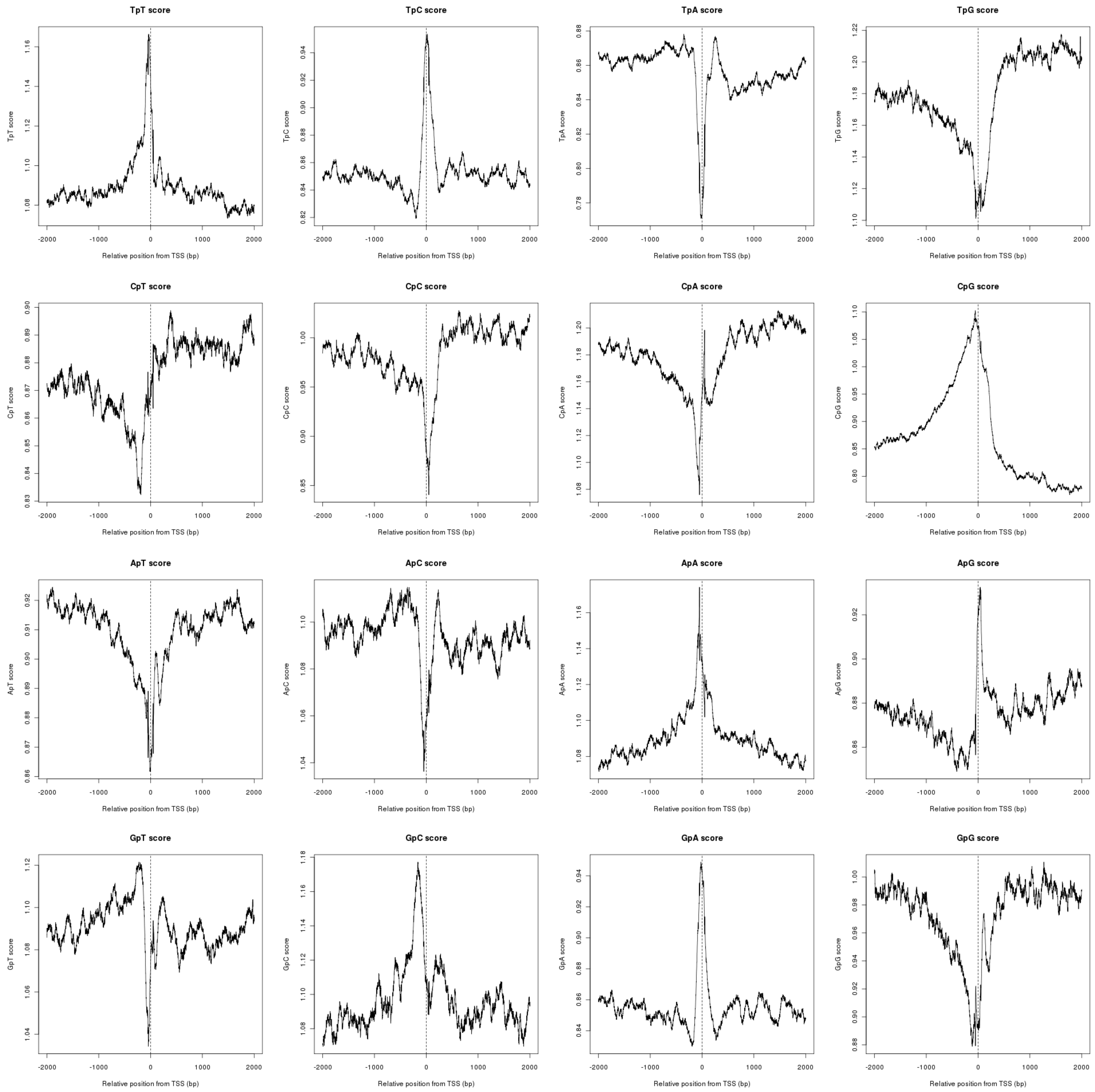

Fig. S2A. Changes in dinucleotide scores in the vicinity of TSSs (ascidian promoters). Note that the vertical scales vary in each panel.

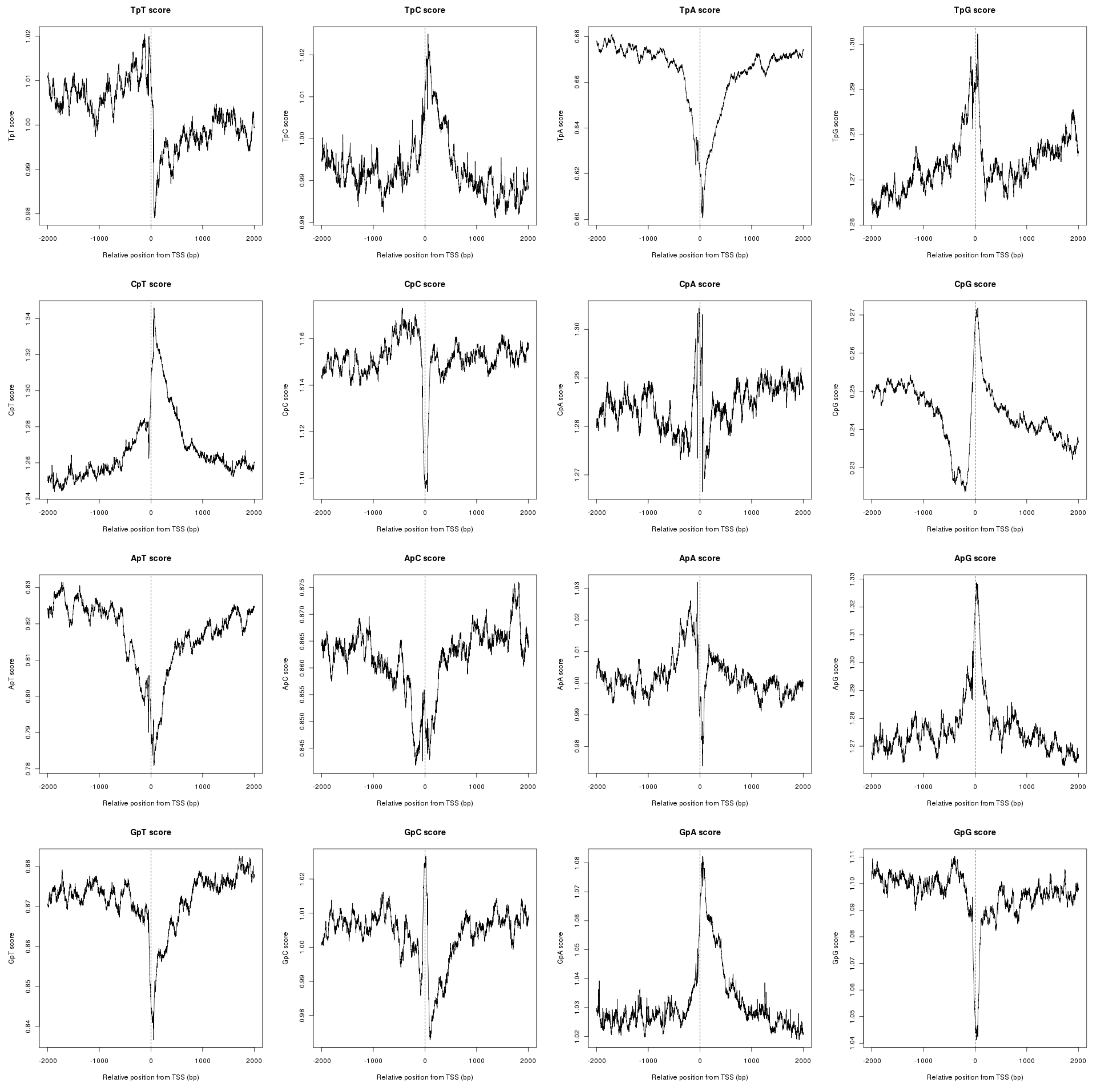

Fig. S2B. Changes in dinucleotide scores in the vicinity of TSSs (human CpG-poor promoters) . Note that the vertical scales vary in each panel.

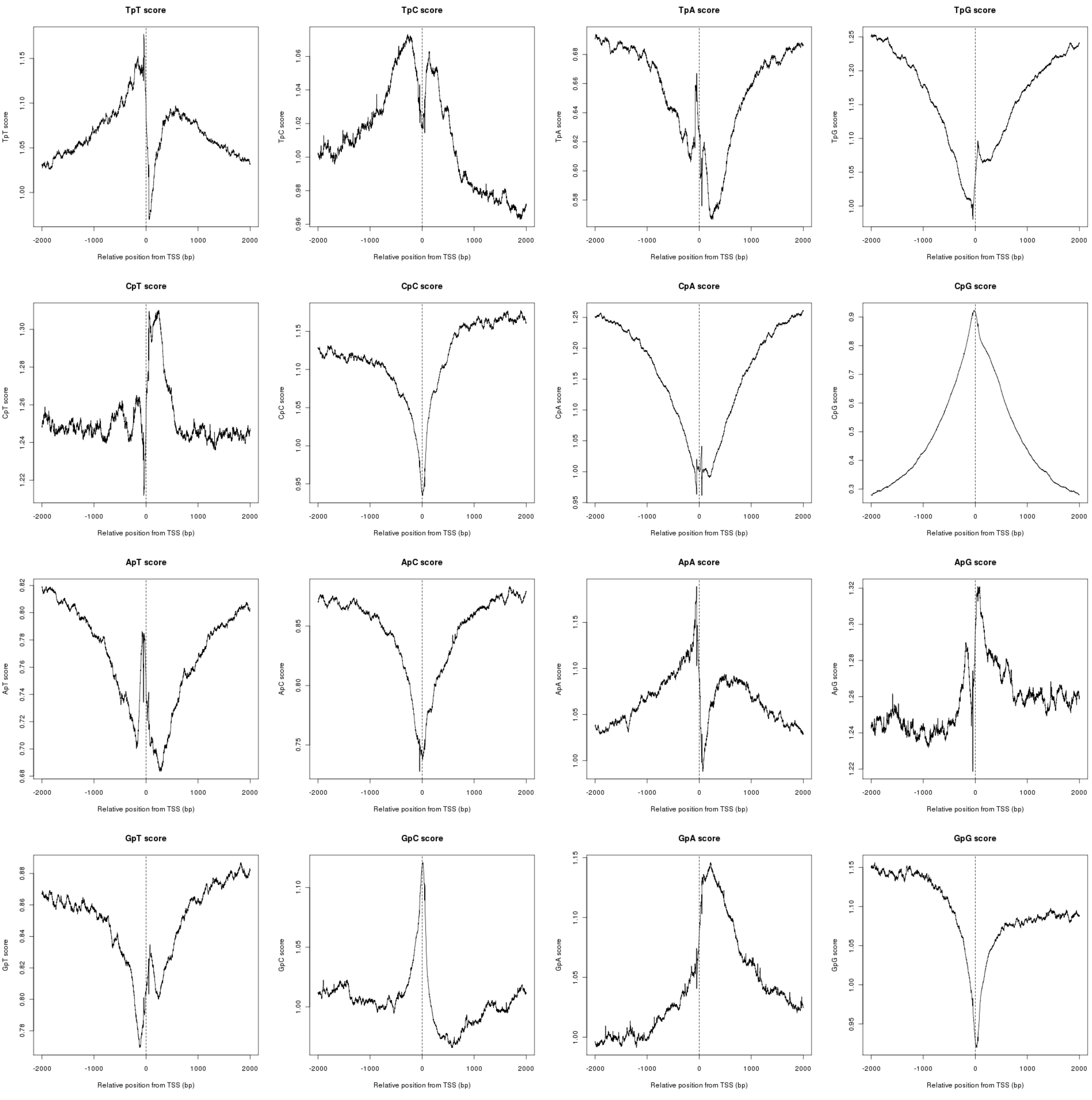

Fig. S2C. Changes in dinucleotide scores in the vicinity of TSSs (human CpG-rich promoters) . Note that the vertical scales vary in each panel.
